# Novel heterocyclic hybrids of pyrazole targeting dihydrofolate reductase: design, biological evaluation and *in* *silico* studies

**DOI:** 10.1080/14756366.2020.1791842

**Published:** 2020-07-16

**Authors:** Ismail M. M. Othman, Mohamed A. M. Gad-Elkareem, Abd El-Galil E. Amr, Mohamed A. Al-Omar, Eman S. Nossier, Elsayed A. Elsayed

**Affiliations:** a Department of Chemistry, Faculty of Science, Al-Azhar University, Assiut, Egypt; b Department of Chemistry, Faculty of Science and Arts of Baljurashi, Albaha University, Saudi Arabia; c Pharmaceutical Chemistry Department, College of Pharmacy, King Saud University, Riyadh, Saudi Arabia; d Applied Organic Chemistry Department, National Research Centre, Giza, Egypt; e Pharmaceutical Medicinal Chemistry Department, Faculty of Pharmacy (Girls), Al-Azhar University, Cairo, Egypt; f Zoology Department, Bioproducts Research Chair, Faculty of Science, King Saud University, Riyadh, Saudi Arabia;; g Chemistry of Natural and Microbial Products Department, National Research Centre, Cairo, Egypt

**Keywords:** Pyrazole, antimicrobial, *in silico* studies, dihydrofolate reductase, molecular docking

## Abstract

A novel series of pyrazole analogues including hydrazones, pyrazolo[4,3-*c*]-pyridazines, pyrazolo[3,4-*e*][1,2,4]triazine and pyrazolo[3,4-*d*][1,2,3]triazoles was designed, synthesised and screened for their *in vitro* antimicrobial and DHFR inhibition activity. Compounds bearing benzenesulphonamide moiety incorporated with 3-methyl-5-oxo-1*H*-pyrazol-4(5*H*)-ylidene) hydrazine **3a** or 6-amino-7-cyano-3-methyl-5*H*-pyrazolo[4,3-*c*]pyridazine **6a** revealed excellent and broad spectrum antimicrobial activity comparable to ciprofloxacin and amphotericin B as positive antibiotic and antifungal controls, respectively. Furthermore, these derivatives proved to be the most active DHFR inhibitors with IC_50_ values 0.11 ± 1.05 and 0.09 ± 0.91 µM, in comparison with methotrexate (IC_50_ = 0.14 ± 1.25 µM). The *in silico* studies were done to calculate the drug-likeness and toxicity risk parameters of the newly synthesised derivatives. Additionally, the high potency of the pyrazole derivatives bearing sulphonamide against DHFR was confirmed with molecular docking and might be used as an optimum lead for further modification.

## Introduction

The increased microbial resistance has led to the demand for new bactericidal and fungicidal agents^1^. The reasons for resistance are the inaccurate diagnosis as well as misuse and widespread use of antimicrobial agents^2^. So, a worldwide effort to search for new generation drugs was stimulated to get new potent, resistance-free, and safer antimicrobial agents^3^^,^^4^.

Recently, dihydrofolate reductase (DHFR) has been considered to be a universal and attractive enzyme which is present in all organisms. Its essential function is to catalyse the reduction of dihydrofolate to tetrahydrofolate within the thymidylate synthesis cycle. As a result, inhibition of DHFR causes “thymineless death”^5–8^. Inhibitors of DHFR explored a crucial role in medicine like methotrexate that is a non-selective inhibitor and a confirmed agent used in oncology for the treatment of rheumatoid arthritis and several cancers^9^. Thus, there are a vast number of interesting target profiles and literatures achievable with DHFR and its inhibitors.

**Figure 1.**
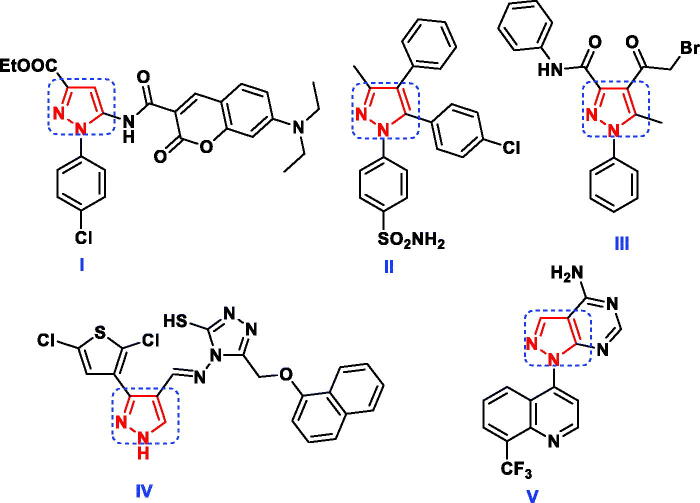
Some reported pyrazole hybrid molecules as antimicrobial agents.

**Figure 2.**
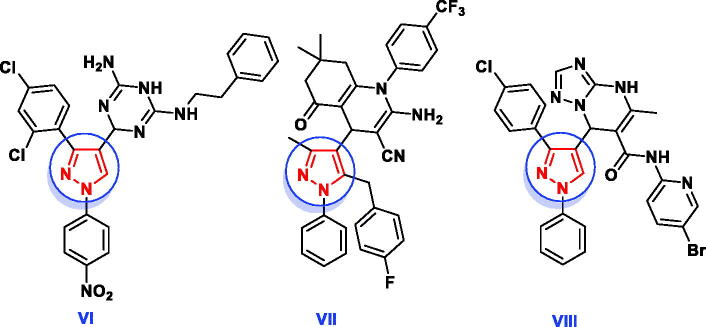
Potent DHFR inhibitors owning pyrazole scaffold as antimicrobial and antimalarial agents.

Pyrazoles are a class of interesting heterocyclic compounds characterised by the presence of two nitrogen atoms adjacent to three carbon atoms in five-membered aromatic ring structure^10^. Pyrazole derivatives play an imperative role in wide spectrum of biological activities including antibacterial^11^, antifungal^12^, anti-inflammatory^13–15^, analgesic^16^, oestrogen receptor binding^17^, neuroprotective^18^, antineoplastic agents^19^. Furthermore, several reports exhibited that pyrazoles **I–IV** and pyrazolo[3,4-*d*]pyrimidine **V** (Figure 1) had high bactericidal and fungicidal activity against the reference strains^20–24^. Additionally, the pyrazole-containing cores (**VI–VII**) were found to be active as antimicrobial and antimalarial through inhibition of DHFR^25–27^ (Figure 2). Regarding to their broad spectrum of biological activities, pyrazole ring has been considered as a favourable unit for addition in the field of drug discovery and therefore in the pharmaceutical industry^10^.

**Figure 3.**
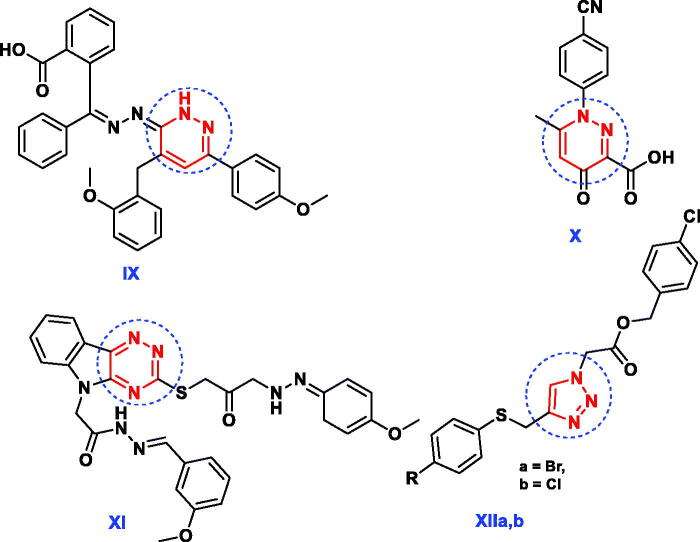
Reported antimicrobial leads containing pyridazine, 1,2,4-triazine and 1,2,3-triazole moieties.

In addition, several pharmacologically active structural units, for example, pyridazines, 1,2,4-triazines and 1,2,3-triazoles are being explored to identify novel lead antimicrobial molecules (Figure 3). The pyridazine derivative **IX** revealed promising *in vitro* antimicrobial activities against Gram + ve and Gram -ve bacteria in comparison with tetracycline^28^. Nagawade *et al.* reported that the pyridazine-3-carboxylic acid analogue **X** showed gratifying *in vitro* antibacterial results approaching that of corresponding reference, ciprofloxacin^29^. Compound containing 1,2,4-triazine nucleus **XI** has been reported to possess better antimicrobial activity with less toxicity than ciprofloxacin^30^. The derivative bearing 1,2,3-triazole scaffold **XIIa** displayed overall encouraging efficiency against all screened microbial strains except *Aspergillus niger*, while the other derivative **XIIb** exhibited better antimicrobial potency against all strains except *Bacillus subtilis* and *Aspergillus niger*^31^.

Motivated by the aforementioned findings, and upon continuation of our research programme in the field of discovery of pyrazole bearing antimicrobial agents^32–34^, some novel pyrazole prototypes were designed and synthesised after exploring molecular fusion with pyridazine, triazine and triazole moieties. The synthesised compounds comprising pyrazole motif were evaluated *in vitro* for their antimicrobial activities against human pathogenic microbes and their inhibitory activities against DHFR enzyme. Finally, molecular docking and *in silico* studies were used to explain the obtained biological data.

## Experimental

### Chemistry

All melting points were determined on a Gallenkamp apparatus and are uncorrected. The IR spectra were measured on a Pye-UnicamSP300 instrument in potassium bromide discs. The ^1^H-NMR and ^13^C-NMR spectra were recorded on Varian Mercury VX (300 MHz) spectrometer (with operating frequencies 300.07 MHz for ^1^H using TMS as an internal standard and 75.45 MHz for ^13^C). Chemical shifts (*δ*) are reported in parts per million (ppm), and coupling constants (*J*) are reported in Hertz (Hz). NMR spectra were recorded at temperature (30–45 °C) and were referenced to the residual signals of DMSO-d_6_. Elemental analyses were carried out by the Micro analytical Centre of Cairo University, Giza, Egypt. The antimicrobial activities were carried out in the Medical Mycology Laboratory of the Regional Centre for Mycology and Biotechnology of Al-Azhar University, Cairo, Egypt.

#### General procedure for preparation of hydrazones 3a and 3b

To a solution of **1** (1 g, 10 mmol) and sodium acetate (1.64 g, 20 mmol ( in absolute ethanol (20 ml), the appropriate diazonium salt of aromatic amines **2a** and **2b** (10 mmol) [prepared by diazotising a solution of each of sulphanilamide and 1-naphthyl amine, respectively (10 mmol) in hydrochloric acid (1.5 ml) with a solution of sodium nitrite (0.69 g, 10 mmol) in 5 ml water in ice bath], was added portion wise with stirring at 0–5 °C. The reaction mixture was stirred for further 1 h and diluted with water. The obtained precipitate was collected by filtration, washed with H_2_O and recrystallised from ethanol to afford the corresponding hydrazine derivatives **3a** and **3b**, respectively.

#### 4-(2-(3-Methyl-5-oxo-1,5-dihydro-4H-pyrazol-4-ylidene)hydrazineyl)benzenesulfonamide (3a)

Yield: 79%; yellow crystals; m.p. 201–203 °C; IR (KBr) *ν* cm^−1^ 3472, 3346 (NH_2_), 3235, 3120 (2NH), 3057 (CH-arom.), 2925 (CH-aliph.), 1733 (CO), 1367, 1145 (SO_2_); ^1^H NMR (DMSO-d_6_) *δ* = 2.61 (s, 3H, CH_3_), 6.44 (s, 2H, NH_2_, exchangeable with D_2_O), 7.19 − 7. 22 (d, *J* = 7.2 Hz, 2H, Ar-H), 7.60 − 7.62 (d, *J* = 7.2 Hz, 2H, Ar-H), 9.15 (*s*, 1H, NH, exchangeable with D_2_O), 12.37 (*s*, 1H, NH-hydrazone); ^13^C NMR (DMSO-d_6_): 12.9, 117.8, 127.4, 130.3, 130.6, 146.1, 149.1, 164.5. Anal. Calcd. for C_10_H_11_N_5_O_3_S (281.29): C, 42.70; H, 3.94; N, 24.90; S, 11.40%. Found: C, 42.51; H, 3.72; N, 24.73; S, 11.75%.

#### 5-Methyl-4–(2-(naphthalene-1-yl)hydrazineylidene)-2,4-dihydro-3H-pyrazol-3-one (3b)

Yield 80%; pale yellow crystals; m.p. 189–191 °C; IR (KBr) *ν* cm^−1^ 3368, 3242 (2NH), 3071 (CH-arom.), 2948 (CH-aliph.), 1730 (CO); ^1^H NMR (DMSO-d_6_) *δ* = 2.48 (*s*, 3H, CH_3_), 7.34 − 7.66 (*m*, 6H, naphthyl-H), 8.18 − 8.21 (d, *J* = 8.1 Hz, 1H, naphthyl-H), 9.16 (*s*, 1H, NH, exchangeable with D_2_O), 12.66 (*s*, 1H, NH-hydrazone, exchangeable with D_2_O); ^13^C NMR (DMSO-d_6_): 14.1, 106.3, 120.2, 122.1, 122.3, 124.2, 125.5, 127.1, 129.4, 131.0, 143.1, 147.2, 182.2. Anal. Calcd. for C_14_H_12_N_4_O (252.27): C, 66.65; H, 4.79; N, 22.21%. Found: C, 66.88; H, 4.57; N, 22.43%.

#### General procedure for preparation of compounds 6a–d

A mixture of **3a** (2.81 g, 10 mmol) with each of either malononitrile (**4a**) (0.66 g, 10 mmol) or ethyl cyanoacetate (**4b**) (1.13 g, 10 mmol) was fused in the presence of ammonium acetate (1.54 g, 20 mmol) at 150 °C for 30 min, then left to stand at room temperature and triturated with absolute ethanol. The solid products so formed were collected by filtration and recrystallised from ethanol/DMF to give **6a** and **6b**, respectively.

Similarly, a mixture of **3b** (2.52 g, 10 mmol) with each of either malononitrile (**4a**) (0.66 g, 10 mmol) or ethyl cyanoacetate (**4b**) (1.13 g, 10 mmol) was fused in the presence of ammonium acetate (1.54 g, 20 mmol) at 150 °C for 30 min, then left to stand at room temperature and triturated with absolute ethanol. The solid products so formed were collected by filtration and recrystallised from ethanol/DMF to give **6c** and **6d**, respectively.

#### 4-(6-Amino-7-cyano-3-methyl-5H-pyrazolo[4,3-c]pyridazin-5-yl)benzenesulphonamide (6a)

Yield: 68%; green crystals; m.p. 298–300 °C; IR (KBr) *ν* cm^−1^ 3441, 3357, 3232, 3125 (2NH_2_), 3054 (CH-arom.), 2933 (CH-aliph.), 2205 (C≡N), 1336, 1174 (SO_2_); ^1^H NMR (DMSO-d_6_) *δ* = 2.77 (*s*, 3H, CH_3_), 6.87 (*s*, 2H, NH_2_, exchangeable with D_2_O), 7.28 − 7.30 (*J* = 7.2 Hz, d, 2H, Ar-H), 8.08 − 8.10 (*J* = 7.2 Hz, d, 2H, Ar-H), 8.69 (*s*, 2H, NH_2_, exchangeable with D_2_O); ^13^C NMR (DMSO-d_6_): 11.1, 82.1, 115.0, 116.1, 129.2, 131.9, 137.8, 140.6, 147.8, 155.3, 162.6. Anal. Calcd. for C_13_H_11_N_7_O_2_S (329.34): C, 47.41; H, 3.37; N, 29.77; S, 9.74%. Found: C, 47.62; H, 3.58; N, 29.54; S, 9.95%.

#### 4-(7-Cyano-3-methyl-6-oxo-1,6-dihydro-5H-pyrazolo[4,3-c]pyridazin-5-yl)benzenesulphonamide (6b)

Yield: 66%; brown crystals; m.p. 294–296 °C; IR (KBr) *ν* cm^−1^ 3437, 3329 (NH_2_), 3241 (NH), 3051 (CH-arom.), 2943 (CH-aliph.), 2207 (C≡N), 1728 (CO), 1341, 1162 (SO_2_); ^1^H NMR (DMSO-d_6_) *δ* = 2.25 (*s*, 3H, CH_3_), 6.72 (*s*, 2H, NH_2_, exchangeable with D_2_O), 7.77 − 7.79 (d, *J* = 7.2 Hz, 2H, Ar-H), 8.10 − 8.13 (d, *J* = 7.2 Hz, 2H, Ar-H), 9.60 (*s*, 1H, NH, exchangeable with D_2_O); ^13^C NMR (DMSO-d_6_): 10.6, 78.3, 115.6, 120.2, 130.5, 134.9, 138.7, 142.3, 148.2, 160.1, 169.7. Anal. Calcd. for C_13_H_10_N_6_O_3_S (330.32): C, 47.27; H, 3.05; N, 25.44; S, 9.71%. Found: C, 47.48; H, 3.28; N, 25.66; S, 9.92%.

#### 6-Amino-3-methyl-5-(naphthalen-1-yl)-5H-pyrazolo[4,3-c]pyridazine-7-carbonitrile (6c)

Yield 65%; brown crystals; m.p. 288–290 °C; IR (KBr) *ν* cm^−1^ 3328, 3232 (NH_2_), 3086 (CH-arom.), 2944 (CH-aliph.), 2215 (C≡N); ^1^H NMR (DMSO-d_6_) *δ* = 2.60 (*s*, 3H, CH_3_) 7.33–7.62 (m, 9H, Ar-H + NH_2_, exchangeable with D_2_O); ^13^C NMR (DMSO-d_6_): 10.1, 99.7, 107.5, 115.0, 118.3, 120.8, 121.0, 125.0, 127.7, 128.5, 129.5, 131.2, 139.7, 141.2, 149.9, 155.5, 162.6. Anal. Calcd. for C_17_H_12_N_6_ (300.32): C, 67.99; H, 4.03; N, 27.98%. Found: C, 67.78; H, 4.23; N, 27.75%.

#### 3-Methyl-5-(naphthalen-1-yl)-6-oxo-5,6-dihydro-1H-pyrazolo[4,3-c]pyridazine-7-carbonitrile (6d)

Yield 61%; brown crystals; m.p. 282–284 °C; IR (KBr) *ν* cm^−1^ 3386 (NH), 3073 (CH-arom.), 2964 (CH-aliph.), 2217 (C≡N), 1732 (CO); ^1^H NMR (DMSO-d_6_) *δ* = 2.47 (*s*, 3H, CH_3_), 7.49–7.78 (*m*, 6H, Ar-H), 8.24 − 8.27 (d, *J* = 8.1 Hz, 1H, naphthyl-H), 9.42 (*s*, 1H, NH, exchangeable with D_2_O); ^13^C NMR (DMSO-d_6_): 12.9, 99.8, 108.1, 115.3, 121.2, 121.6, 123.7, 16.6, 126.9, 127.8, 129.3, 130.8, 138.7, 142.2, 146.1, 162.0, 168.7. Anal. Calcd. for C_17_H_11_N_5_O (301.30): C, 67.77; H, 3.68; N, 23.24%. Found: C, 67.56; H, 3.89; N, 23.47%.

#### General procedure for preparation of compounds 8a and 8b

Dissolve either (2.81 g, 10 mmol) of **3a** or (2.52 g, 10 mmol) of **3b** in absolute ethanol (30 ml), and then add 0.5 ml of triethylamine. Each of the solutions was treated with phenylisothiocyanate (1.35 g, 10 mmol). The reaction mixtures were heated under reflux for 8 h then cooled. The so formed precipitate during heating in each case was collected by filtration and recrystallised from dioxane to give **8a** and **8b**, respectively.

#### 4-(7-Methyl-4-phenyl-3-thioxo-3,4-dihydro-2H-pyrazolo[3,4-e][1,2,4]triazin-2-yl)benzenesulphonamide (8a)

Yield: 59%; light yellow crystals; m.p. 275–277 °C; IR (KBr) *ν* cm^−1^ 3322, 3268 (NH_2_), 3080 (CH-arom.), 2953 (CH-aliph.), 1386, 1182 (SO_2_), 1317 (CS); ^1^H NMR (DMSO-d_6_) *δ* = 2.28 (*s*, 3H, CH_3_), 6.25 (*s*, 2H, NH_2_, exchangeable with D_2_O), 6.77–8.06 (*m*, 9H, Ar-H); ^13^C NMR (DMSO-d_6_): 21.6, 124.2, 127.11, 127.14, 129.4, 132.0, 134.5, 136.2, 137.9, 143.5, 151.4, 157.6, 176.1. Anal. Calcd. for C_17_H_14_N_6_O_2_S_2_ (398.46): C, 51.24; H, 3.54; N, 21.09; S, 16.09%. Found: C, 51.55; H, 3.75; N, 21.32; S, 16.30%.

#### 7-Methyl-2-(naphthalen-1-yl)-4-phenyl-2,4-dihydro-3H-pyrazolo[3,4-e][1,2,4]triazine-3-thione (8b)

Yield 56%; light yellow crystals; m.p. 257–259 °C; IR (KBr) *ν* cm^−1^ 3066 (CH-arom.), 2941 (CH-aliph.), 1278 (CS); ^1^H NMR (DMSO-d_6_) *δ* = 2.48 (*s*, 3H, CH_3_), 7.61–7.88 (*m*, 11H, Ar-H), 8.11 − 8.14 (d, *J* = 8.1 Hz, 1H, naphthyl-H); ^13^C NMR (DMSO-d_6_): 20.0, 112.1, 118.2, 123.1, 123.3, 124.9, 126.2, 127.1, 127.7, 129.0, 129.8, 133.1, 134.9, 135.0, 136.8, 137.0, 140.6, 151.0, 157.6, 174.1. Anal. Calcd. for C_21_H_15_N_5_S (369.44): C, 68.27; H, 4.09; N, 18.96%. Found: C, 68.48; H, 4.30; N, 18.74%

#### General procedure for preparation of compounds 9a and 9b

Dissolve either (2.81 g, 10 mmol) of **3a** or (2.52 g, 10 mmol) of **3b** in dimethylformamide (30 ml), and then add 0.5 ml of triethylamine. Each of the solutions was treated with hydroxylamine hydrochloride (0.69 g, 10 mmol). The reaction mixtures were heated under reflux for 24 h then left to cool, and poured into crushed ice and acidified with 10% HCl. The solid product obtained was collected by filtration and recrystallised from dioxane/ethanol to give **9a** and **9b**, respectively.

#### 4-(6-Methylpyrazolo[3,4-d][1,2,3]triazol-2(4H)-yl)benzenesulphonamide (9a)

Yield: 63%; pale yellow crystals; m.p. > 300 °C;IR (KBr) *ν* cm^−1^ 3381, 3257 (NH_2_), 3136 (NH), 3077 (CH-arom.), 2925 (CH-aliph.), 1385, 1131 (SO_2_); ^1^H NMR (DMSO-d_6_) *δ* = 2.89 (s, 3H, CH_3_), 6.31(*s*, 2H, NH_2_, exchangeable with D_2_O), 7.54 − 7.57 (d, *J* = 7.2 Hz, 2H, Ar-H), 7.64 − 7.66 (d, *J* = 7.2 Hz, 2H, Ar-H), 12.62 (*s*, 1H, NH, exchangeable with D_2_O); ^13^C NMR (DMSO-d_6_): 10.2, 127.8, 129.4, 134.2, 141.9, 143.9, 146.1. Anal. Calcd. for C_10_H_10_N_6_O_2_S (278.29): C, 43.16; H, 3.62; N, 30.20; S, 11.52%. Found: C, 43.37; H, 3.84; N, 30.41; S, 11.75%.

#### 6-Methyl-2-(naphthalen-1-yl)-2,4-dihydropyrazolo[3,4-d][1,2,3]triazole (9b)

Yield: 67%; pale yellow crystals; m.p. > 300 °C; IR (KBr) *ν* cm^−1^ 3292 (NH), 3086 (CH-arom.), 2944 (CH-aliph.); ^1^H NMR (DMSO-d_6_) *δ* = 2.72 (*s*, 3H, CH_3_) 7.40–8.20 (*m*, 7H, Ar-H), 12.76 (*s*, 1H, NH, exchangeable with D_2_O); ^13^C NMR (DMSO-d_6_): 10.3, 123.2, 123.5, 125.5, 125.9, 127.3, 129.8, 130.6, 132.1, 134.5, 139.1, 146.1. Anal. Calcd. for C_14_H_11_N_5_ (249.27): C, 67.46; H, 4.45; N, 28.10%. Found: C, 67.46; H, 4.45; N, 28.10%.

### Biological activity

#### Antimicrobial activity sensitivity assay

The target compounds **3**–**9** were screened *in vitro* opposite to various types of bacteria, *Streptococcus pneumoniae* and *Bacillus subtilis* as examples of Gram-positive bacteria, and *Pseudomonas aeruginosa* and *Escherichia coli* as examples of Gram-negative bacteria and for their Antifungal activities against *Aspergillus fumigates* and *Candida albicans*, respectively. Solutions of concentrations (1 µg/mL) of the target compounds were used. The agar media were inoculated with different microorganism’s culture tested after 24 h of inoculation at 37 °C for bacteria and for antifungal tested after 72 h of inoculation at 28 °C. Ciprofloxacin and amphotericin B were used as standard antibacterial and antifungal drugs, respectively. The diameter of inhibition zone (mm) was measured for the biologically activity using the diffusion technique^35^. The active compounds **3a**, **3b**, **6a**, **6b**, **8a** and **9a** were further investigated to determine their antimicrobial activity expressed in terms of minimum inhibitory concentration (MIC) using the modified agar well diffusion method that mentioned above. The different concentrations (triplicate) of each compound were tested and compared with standard drugs.

#### Dihydrofolate reductase (DHFR) inhibition

The newly synthesised active targets **3a**, **3b**, **6a**, **6b**, **8a** and **9a** were assessed for their *in vitro* inhibition against dihydrofolate reductase (DHFR) in confirmatory diagnostic unit, Vacsera, Egypt. Methotrexate was used as a reference drug following the previously mentioned method^36^. The obtained results are depicted as IC_50_ values of enzyme inhibition in Table 2. The assay mixture contained 50 µM Tris–HCl buffer (pH 7.4), 50 µM NADPH, 10 µl DMSO or the same volume of DMSO solution containing the test compounds to a final concentration of 10^−11^ to 10^−5 ^M, and 10 µl of DHFR, in a final volume of 1.0 ml. After addition of the enzyme, the mixture was incubated at room temperature for 2.0 min, and the reaction was initiated by adding 5 µl of dihydrofolic acid, the change in absorbance (ΔOD/min) was measured by the spectrophotometer at 340 nm and 22 °C, kinetic programme (reading every 15 s for 2.5 min). Results are reported as % inhibition of enzymatic activity calculated using the following formula:

Fractional activity of enzyme=(Sample ΔOD/min − blank ΔOD/min)x × d/12 :3x×Vx×mg P/mL

where ΔOD/min: the spectrophotometer readings 12.3: extinction coefficient for the DHFR reaction at 340 nm. V: Enzyme volume in mL (the volume of enzyme used in the assay) d: The dilution factor of the enzyme sample. mgP/mL: enzyme concentration of the original sample before dilution.

### *In silico* calculations of molecular properties

Molecular descriptors express the pharmacokinetic, pharmacodynamic and physicochemical effects of all newly synthesised targets **3**, **6**, **8** and **9**. The lipophilicity (milogP) and topological polar surface area (tPSA) were measured using the online Molinspiration software^37^ while the drug score, drug-likeness and aqueous solubility were evaluated *via* the OSIRIS property explorer software^38^. Furthermore, good bioavailability is more preferable for targets having TPSA of ≤140 Å^2^ and ≤10 rotatable bonds^39^.

### Molecular modelling study

The molecular docking a powerful tool to understanding and rationalise the obtained biological results. The interactions of the newly synthesised compounds **3a** and **6a** having the highest DHFR inhibitory activity were investigated with the active site of the target kinase to study their mode of binding and orientations. All the molecular modelling studies were carried out using MOE, 10.2008 software^40^^,^^41^. All minimisations were performed with MOE until an RMSD gradient of 0.05 kcal.mol^−1 ^Å^−1^ with MMFF94x force field and the partial charges were automatically calculated. The co-crystallised structure of the dihydrofolate reductase enzyme was downloaded from Protein Data Bank website (PDB ID: 1DLS)^36^. The enzyme structure was prepared for molecular docking using Protonate 3D protocol in MOE with the default options. The co-crystallised inhibitor was used to define the active site for molecular docking. Triangle Matcher placement method and London dG scoring function were used in the docking protocol. Docking setup was first validated by self-docking of the co-crystallised inhibitor in the enzyme active site giving a docking pose with an energy score (S) = −11.68 kcal/mol and an RMSD of 0.88 Å from the co-crystalised ligand pose (Figure 5). Then, the validated molecular docking setup was used to investigate the ligand–target interactions of the newly synthesised compounds **3a** and **6a** in the DHFR active site to predict their binding pattern and to investigate their ability to satisfy the required structural features for binding interactions (Figures 6 and 7).

## Results and discussion

### Chemistry

**Scheme 1.**
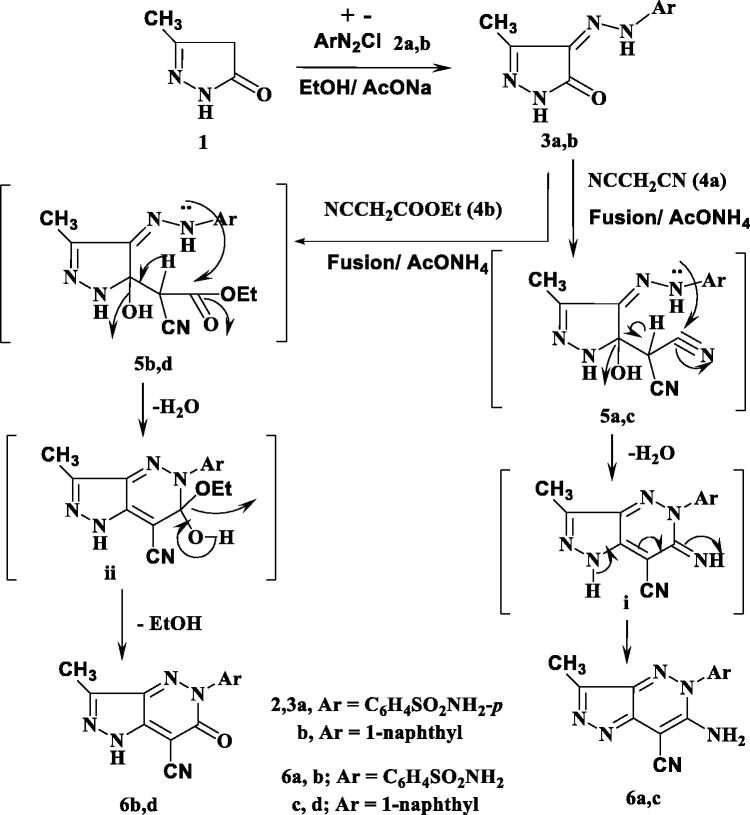
Synthesis of pyrazolopyridazine derivatives.

**Scheme 2.**
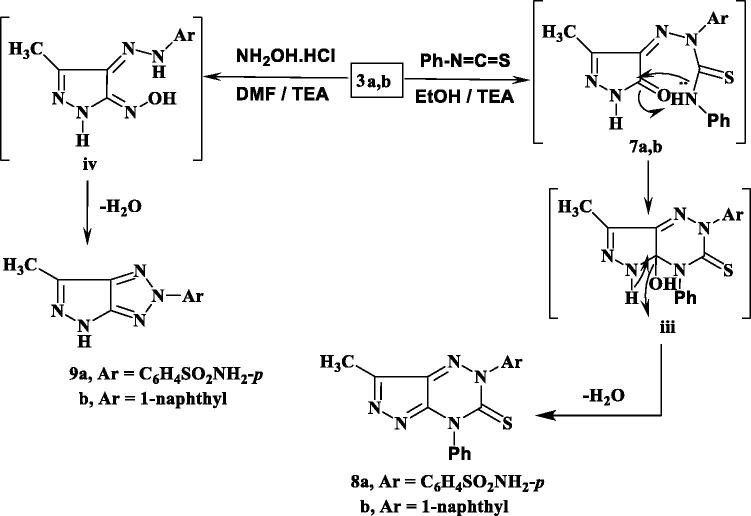
Synthesis of pyrazolotriazine and pyrazolotriazole derivatives.

The synthetic strategies approved for the synthesis of the intermediate and target compounds are outlined in Schemes 1 and 2. Thus, 3-methylpyrazol-5(4*H*)-one **1** was coupled smoothly with diazonium salts **2a** and **2b** in the presence of ethanol and sodium acetate at 0–5 °C, to afford the respective hydrazines **3a** and **3b** (Scheme 1). The latter products were established on the basis of their analytical and spectral data. The ^1^H NMR spectrum of **3a**, as an example, revealed the two signals at δ 9.15 and 12.37 ppm due to two NH groups, in addition to the presence of amino, methyl and aromatic protons. While, its ^13^C NMR spectrum revealed the signals at *δ* 12.9 and 164.5 ppm assigned to the carbons of the CH_3_ and CO groups, (See Scheme 1 and Experimental part). Moreover, the obtained arylhydrazino derivatives have been utilised as starting materials for preparing the targeted pyrazolopyridazine ring system. Fusion of hydrazines **3a** and **3b** with malononitrile (**4a**) in the presence of ammonium acetate over melting point afforded the 6-aminopyrazolo [4,3-*c*]-pyridazine derivatives **6a** and **6c**. The structures of **6a** and **6c** were established via inspection of their spectral data. For example, the infra-red of **6a** indicated the presence of the two NH_2_ and C≡N absorption band at ν = 3441, 3357, 3232, 3125 and 2205 cm^−1^. While, its ^1^H NMR revealed singlet signal at *δ* 2.77 ppm which was assigned for CH_3_ group, two signals at *δ* 6.87 and 8.69 due to two NH_2_ protons and the absence of any signals may be attributed to NH protons, beside aromatic protons in the molecule. The ^13^CNMR of **6a** showed signals at 11.1and 115.0 for the carbons of the CH_3_, and C≡N groups, in addition to the sp_2_ carbon atoms (Scheme 1 and Experimental part). The formation of **6a** and **6c** from **3a** and **3b** is assumed to proceed through the intermediate of non-isolable adducts **5a** and **5c,** which cyclised into the imino structure **i** via loss of water molecule, which tautomerised into the end amino products **6a** and **6c** (Scheme 1).

Similarly, **3a** and **3b** reacted with ethyl cyanoacetate (**4b**) in the same above reaction conditions to afford 6-oxopyrazolo[4,3-*c*]pyridazine derivatives **6b** and **6d** based on the elemental analysis and spectral data. The IR spectra of **6b** and **6d** indicate the presence of C≡N absorption bands at 2207, 2217 cm^−1^, respectively and C=O at 1728, 1732 cm^−1^, respectively. ^1^H NMR spectra of **6b** and **6d** showed the presence of only one signal attributed to NH at 9.60 and 9.42 ppm, respectively. Also, its ^13^C NMR spectra revealed the signals of amidic C=O at 169.7 and 168.7 ppm, respectively.

The structure **6b** and **6d** was assumed to be formed through the non-isolable adducts **5b** and **5d**, which underwent cyclisation into structure **ii** via loss of water molecule then loss of ethanol molecule to give the end reaction products the oxo derivatives **6b** and **6d** (Scheme 1).

The starting material **3a** and **3b** used in this study was proved to be a versatile for synthesis of some novel pyrazolo[3,4-*e*][1,2,4]triazines and pyrazolo[3,4-*d*]-[1,2,3]triazoles.

Thus, **3a** and **3b** were reacted with phenylisothiocyanate in refluxing ethanol containing a catalytic amount of triethylamine to give the pyrazolotriazine derivatives **8a** and **8b**. The composition and structure of products **8a** and **8b** were established by the results of analytical and compatible spectroscopic data (IR, ^1^H NMR and ^13^C NMR), (Scheme 2 and Experimental part).

The infra-red spectra of **8a** and **8b** indicated the absence of the 2NH and carbonyl absorption bands and revealed the characteristic absorption band for the thiocarbonyl group. For example, the ^1^H NMR spectrum of **8a** in DMSO-d_6_ revealed two singlets at *δ* 2.28, 6.25 ppm which were assigned for methyl and NH_2_ protons, respectively, and disappearance of any signal attributable to the NH proton, in addition to the presence of aromatic protons. Also, ^13^C NMR spectrum showed confirmatory signal of the CS group around *δ* 176.1 ppm. Formation of **8** was proceeded via the nucleophilic addition of **3** to phenyl isothiocyanate to give the non-isolable adduct **7** followed by cyclisation through the nucleophilic attack of N atom to the carbon of the C = O group giving structure **iii** which upon elimination of H_2_O molecule afforded the end reaction products **8a** and **8b**.

Further illustration of the hydrazone structure **3** came from the reaction with hydroxyl amine hydrochloride in the presence of dimethyl formamide (DMF)/triethylamine solution under reflux to give the final product pyrazolotriazole derivative **9**. The structures of **9a** and **9b** were in consistence with their respective analytical and spectral studies (Scheme 2).

The IR spectrum of **9a**, taken as a typical example, was characterised by the disappearance of absorption band due to the CO group. Its ^1^HNMR showed two singlet at *δ* 2.89, 6.31 ppm which were assigned for CH_3_ and NH_2_ protons, respectively and a singlet at *δ* 12.62 ppm attributable to the pyrazole-NH proton, in addition to the presence of aromatic protons (cf. Scheme 2 and Experimental Section). The carbonyl group of pyrazole moiety was also absent in ^13^C-NMR spectra.

### Biological activity

#### Antimicrobial sensitivity assay

**Figure 4.**
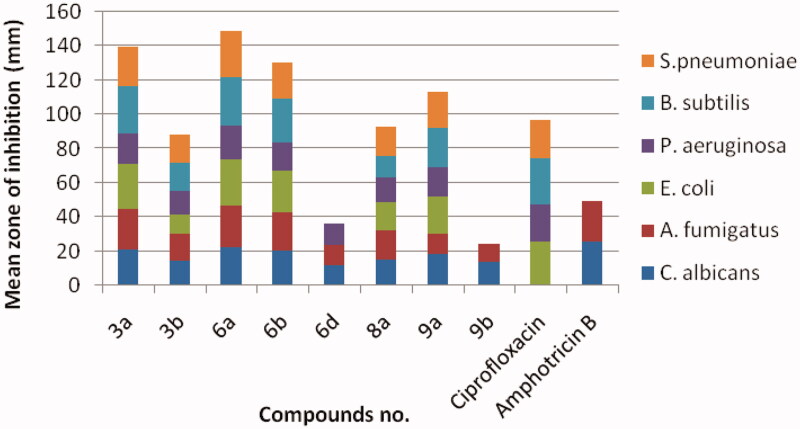
Antimicrobial activity of the most active compounds against different bacterial and fungal strains compared with the reference drugs, ciprofloxacin and amphotericin B, respectively.

The antimicrobial activity of all synthesised compounds **3**, **6**, **8** and **9** was evaluated by agar well diffusion method^35^ using ciprofloxacin and amphotericin B as standard references for antibacterial and antifungal activity, respectively. The screening was done for the *in vitro* antibacterial activity against Gram-positive *Streptococcus pneumoniae* and *Bacillus subtilis*, and Gram-negative *Pseudomonas aeruginosa and Escherichia coli*, and for the *in vitro* antifungal activity against *Aspergillus fumigates* and *Candida albicans*. The measure zones of inhibition are presented in Figure 4 in mm. By investigation of the given data, it was observed that compounds **3a**, **3b**, **6a**, **6b**, **8a** and **9a** displayed excellent inhibition zone diameter ranging from 11.6 to 28.7 mm against all tested strains in comparison with the reference drugs. On the other hand, the remaining derivatives showed weak or no antimicrobial activity.

Minimum inhibitory concentration (MIC) of the most active compounds **3a**, **3b**, **6a**, **6b**, **8a** and **9a** was measured *in vitro* using twofold serial dilution technique^35^. The results of MIC were recorded in Table 1. It was noticed that compound **6a** among other derivatives revealed excellent and the highest MIC value all over the tested strains. Moreover, the derivatives **3a** and **6b** were equipotent with the reference, amphotericin B against *A.* *fumigates* (MIC = 1.95 µg/mL) and exhibited two folds decrease in the potency than the standard, ciprofloxacin against *S. pneumonia* (MIC = 1.95 and 0.98 µg/mL, respectively). Additionally, **6b** was equipotent in the antifungal activity with reference against *A.* *fumigates*. All the remaining derivatives illustrated from moderate-to-weak antimicrobial activity.

**Table 1.** Minimal inhibitory concentrations (MICs) of the synthesised compounds against the tested pathogenic bacteria and fungi.^a^

Compound No.	MIC (Mean ± SEM) (μg/mL)					
	Gram-positive Bacteria		Gram-negative Bacteria		Fungi	
	*S. pneumonia* *RCMB 010010*	*B. subtilis* *RCMB 010067*	*P. aeruginosa* *RCMB 010043*	*E. coli RCMB 010052*	*A. fumigatus* *RCMB 002568*	*C. albicans* *RCMB 005036*
**3a**	1.95 ± 0.3	0.98 ± 0.1	7.81 ± 0.3	0.98 ± 0.6	1.95 ± 0.1	3.9 ± 0.3
**3b**	7.81 ± 0.1	15.63 ± 0.5	125 ± 0.3	250 ± 0.5	125 ± 0.2	62.5 ± 0.1
**6a**	0.98 ± 0.1	0.49 ± 0.2	3.9 ± 0.1	0.49 ± 0.4	0.98 ± 0.2	3.9 ± 0.03
**6b**	1.95 ± 0.4	0.98 ± 0.1	62.5 ± 0.3	0.98 ± 0.1	1.95 ± 0.3	7.81 ± 0.05
**8a**	15.63 ± 0.5	125 ± 0.4	125 ± 0.3	62.5 ± 0.3	3.9 ± 0.1	62.5 ± 0.5
**9a**	3.9 ± 0.3	1.95 ± 0.08	15.63 ± 0.5	1.95 ± 0.3	250 ± 0.2	7.81 ± 0.2
**Ciprofloxacin**	0.98 ± 0.2	0.1 ± 0.3	0.5 ± 0.05	0.01 ± 0.1	–	–
**AmphotricinB**	–	–	–	–	1.95 ± 0.3	3.9 ± 0.1

–: Not tested; SEM: standard error mean; each value is the mean of three values.

^a^Antibacterial and antifungal activities were expressed as MIC in µg/mL.

#### Structure–activity relationship (SAR) study

By inspection of the previous data, it was clear that all derivatives having benzene sulphonamide moiety showed higher and remarkable antimicrobial activity than those having naphthalenyl one. Incorporation of 3-methyl-5-oxopyrazoline with hydrazinyl open chain at p-4 displayed noticeable and excellent antimicrobial activity especially against *S. pneumonia*, *A.* *fumigates* and *C. albicans* in compound **3a**. Fusion of the 3-methyl-5-oxopyrazoline with pyridazine moiety giving 6-amino-7-cyano-3-methyl-5*H*-pyrazolo[4,3-*c*]pyridazine improved the potency to the highest value in **6a** against almost strains in comparison with references. However, **6b** revealed approximately the same excellent potency of **3a**. Replacement of pyridazine with triazine or triazole moieties caused drop in the antimicrobial activity in **8a** and **9a**, respectively.

#### *In vitro* enzyme assay on dihydrofolate reductase (DHFR)

The inhibitory activity of the synthesised compounds **3a**, **3b**, **6a**, **6b**, **8a** and **9a** were examined against DHFR using reported procedure^36^. The results of the inhibitory activity are shown as IC_50_ values in Table 2 using methotrexate as a positive control. As expressed in Table 2, compounds **3a** and **6a** proved to be the highest active inhibitors with IC_50_ values 0.11 ± 1.05 and 0.09 ± 0.91 µM, in comparison with Methotrexate (IC_50_ = 0.14 ± 1.25 µM). However, the rest derivatives revealed moderate inhibitory activities via **8a** and **9a** (IC_50_ = 1.36 ± 0.12 and 1.45 ± 0.23 µM, respectively) and weak ones through **3b** and **6b** (IC_50_ = 18.30 ± 0.81 and 5.24 ± 0.37 µM, respectively).

**Table 2.** *In vitro* inhibitory activities of the screened compounds **3a**, **3b**, **6a**, **6b**, **8a** and **9a** against DHFR enzyme.

Compound No.		IC_50_ (Mean ± SEM) (µM)
		DHFR
**3a**		0.11 ± 1.05
**3b**		18.30 ± 0.81
**6a**		0.09 ± 0.91
**6b**		5.24 ± 0.37
**8a**		1.36 ± 0.12
**9a**		1.45 ± 0.23
**Methotrexate**		0.14 ± 1.25

IC_50_: Compound concentration required to inhibit DHFR enzyme activity by 50%; SEM: standard error mean; each value is the mean of three values.

Correlation between the chemical structure and the inhibitory activity of the screened derivatives over DHFR enzyme revealed that the existence of benzene sulphonamide moiety in **3a**, **6a, 8a and 9a** led to enhanced activity, and the potency order was **6a** > **3a** > **8a** > **9a**. Moreover, the attachment of pyrazoline ring with hydrazinyl group in **3a** or hybridisation with pyridazine in **6a** possessed better inhibitory activity than with triazine or triazole in **8a** and **9a.**

### *In silico* calculations of molecular properties

#### Drug-likeness parameters

A molecular property is a complex balance of various structural features which determine whether a specific molecule is similar to the known drugs and to facilitate the drug-likeness of the candidate drug. Therefore, the calculated molecular descriptors of the new compounds expressed in terms of calculating log P, molecular size, flexibility and the presence of hydrogen-donor and acceptors using Molinspiration tool^37^ and the results are depicted in Table 3.

**Table 3.** Calculated molecular properties of the synthesised compounds **3**, **6**, **8** and **9** for assessment of the drug likeness.

Compound No. Rule	m_i_LogP^a^ <5	% ABS^b^	TPSA^c^	N_atoms_^d^	MW^e^ <500	M.Vol.^f^	n_ON_^g^ <10	n_OHNH_^h^ <5	n_viol_.^i^	n_rotb._^j^ (<10)
**3a**	0.07	64.04	130.31	19	281.30	222.79	8	4	0	3
**3b**	2.54	84.80	70.14	19	252.28	224.06	5	2	0	2
**6a**	−0.04	56.01	153.59	23	329.35	259.77	9	4	0	2
**6b**	−0.20	58.09	147.54	23	330.33	256.61	9	3	0	2
**6c**	2.63	76.77	93.42	23	300.32	261.04	6	2	0	1
**6d**	2.48	78.85	87.37	23	301.31	257.88	6	1	0	1
**8a**	1.27	71.49	108.71	27	398.47	316.26	8	2	0	3
**8b**	3.94	92.25	48.54	27	369.45	317.53	5	0	0	2
**9a**	0.03	67.74	119.57	19	278.30	216.61	8	3	0	2
**9b**	2.70	88.50	59.40	19	249.28	217.88	5	1	0	1
**Ciprofloxacin**	−0.70	83.27	74.57	24	331.35	285.46	6	2	0	3
**Amphotericin B**	−2.49	1.27	319.61	65	924.09	865.48	18	13	3	3

^a^Octanol–water partition coefficient, calculated by the methodology developed by Molinspiration.

^b^% ABS: percentage of absorption.

^c^TPSA: topological polar surface area.

^d^Number of non-hydrogen atoms.

^e^Molecular weight.

^f^Molecular volume.

^g^Number of hydrogen-bond acceptors (O and N atoms).

^h^Number of hydrogen-bond donors (OH and NH groups).s

^i^Number of “Rule of five” violations.

^j^Number of rotatable bonds.

By analysing the obtained data, it can be concluded that there are linear correlations between the lipophilicity and the activity of the new derivatives and all of them are fully in agreement with the Lipinski’s rule. All the tested compounds have the number of rotatable bonds in the range of 1–3, with the values ranging from 2 into 3 allowed to the most bioactive compounds **3a**, **3b**, **6a**, **6b**, **8a** and **9a** and therefore, obviously exhibiting small conformational flexibility. Results shown in Table 3 indicated that all of the analysed compounds have Topological polar surface area (TPSA) values **˂**140 Å^2^ except for compounds **6a** and **6b** have values 153.59 and 147.54, respectively, and therefore are candidate for good solubility, capacity for penetrating cell membranes and intestinal absorption. Compounds with n_OHNH_ value (H-bond donors) less than 5 exhibited increased solubility in cellular membranes and all the test compounds have n_OHNH_ value in the range of 0–4 which is less than 5. Compounds have n_ON_ value (H-bond acceptors) in the range of 5–9 and molecular weight in the range of 249.28–398.47. Molecular volumes of the compounds in the series increased as increasing MW and ranged from 216.61 to 317.53. The partition coefficient is vital to examine the physico-chemical properties associated with biological activity. The milogP values for all compounds under investigation are less than 5, outlining good lipophilicity (as defined by the pH-partition hypothesis) with various possible biological sites reasonable oral absorption and lower permeation across biological membranes but higher aqueous solubility especially for compounds **3a**, **6a**, **6b**, **8a** and **9a** that are most bioactive compounds. Higher solubility is a good factor in drug formation and might generally be reduced in more highly hydrophobic compounds. The greater lipophilicity of these compounds may protect against ROS damage and is due in part to their smaller polar surface area which is another useful descriptor of the oral bioavailability and drug transport properties. As can be seen, compounds **3b** and **6c** that contains the nonpolar naphthyl substituent exhibited greater lipophilicity (milogP = 2.54 and 2.63, respectively) as compared to compound **3a** and **6a** (milogP = 0.07 and −0.04) with polar group (sulphonamide). Also, the same phenomena were observed in compounds **6d, 8b, 9b,** that contains naphthyl substituent exhibited greater lipophilicity (milogP = 2.48, 3.94, 2.70, respectively) as compared to compounds **6b, 8a, 9a** (milogP = −0.02, 1.27, 0.03) with polar group (sulphonamide). Our study revealed that compounds **6c, 6d, 8b** and **9b** (greater lipophilicity) are biologically either inactive or less active, but compounds **6a, 6b, 8a** and **9a** (least lipophilicity) are highly active against the tested bacteria and fungi, while compound **3a** are highly active than **3b**. Our results exhibited that there is a clear relationship between lipophilicity and antimicrobial activity. The most active compounds against all microbial strains were **3a** (milogP = 0.07), **6a** (milogP = −0.04), **6b** (milogP = −0.02), **8a** (milogP = 1.27) and **9a** (milogP = 0.03). In the light of the above results, we achieved that all tested compounds satisfy the “Rule of five” and meet all criteria for good permeability and bioavailability.

#### Toxicity risks

Toxicity risks and physicochemical properties of the newly prepared compounds were evaluated through the methodology developed by Osiris^38^. The data given in Table 4, displayed that all compounds are expected to be non-irritating and high risk of reproductive effects. Also, compounds **3b, 6b, 6d, 8b** and **9b** have shown the non-mutagenic and non-tumorigenic effects. The aqueous solubility of a compound significantly affects its absorption and distribution properties. It is well known that more than 80% of the drugs on the market have estimated solubility values greater than −4. From Table 4, it was noted that compounds **3a**, **3b**, **6b**, **9a** and **9b** exhibited solubility values above −4 and they are expected to have good aqueous solubility which significantly affects their absorption and distribution characteristics. Osiris programme was used for calculating the fragment-based drug-likeness of the synthesised compounds; a positive value indicates that the designed molecule contains fragments that are frequently present in commercial drugs. The results indicated that all target compounds have drug-likeness values except **6c** and **6d** in the comparable zone with that of the standard drugs. The drug score combines drug likeness, miLogP, solubility, molecular weight, and toxicity risks in one handy value that may be used to judge the compound’s overall potential to qualify for a drug^39^. A value of 0.5 or more makes the compound a promising lead for future development of safe and efficient drugs. The overall drug score values for the synthesised compounds were calculated and compared to those of the standard drugs. Compounds **3a**, **6b** and **9a** own good drug score values (Table 4).

**Table 4.** Toxicity risks, solubility, drug-likeness, and drug score of the synthesised compounds.

	Toxicity risks				Solubility	Drug likeness	Drug Score
Comp. no.	Mutagenicity	Tumorigenicity	Irritancy	Reproductive effect			
3a	green	green	green	red	−2.09	6.32	0.56
**3b**	red	red	green	red	−3.8	4.81	0.17
**6a**	green	green	green	red	−4.15	0.33	0.38
**6b**	green	green	green	red	−2.79	1.59	0.49
**6c**	Red	red	green	red	−5.86	−1.42	0.07
**6d**	Red	red	green	red	−4.5	−0.01	0.12
**8a**	Red	green	green	red	−4.4	4.87	0.24
**8b**	Red	red	green	red	−6.11	3.16	0.08
**9a**	Green	green	green	red	−2.22	4.26	0.56
**9b**	Red	red	green	red	−3.93	2.52	0.17
**Ciprofloxacin**	Green	green	green	green	−3.32	2.07	0.82
**Amphotericin B**	Green	green	green	green	−5.08	−0.14	0.27

Red: high risk; green: low risk.

### Molecular modelling study

**Figure 5.**
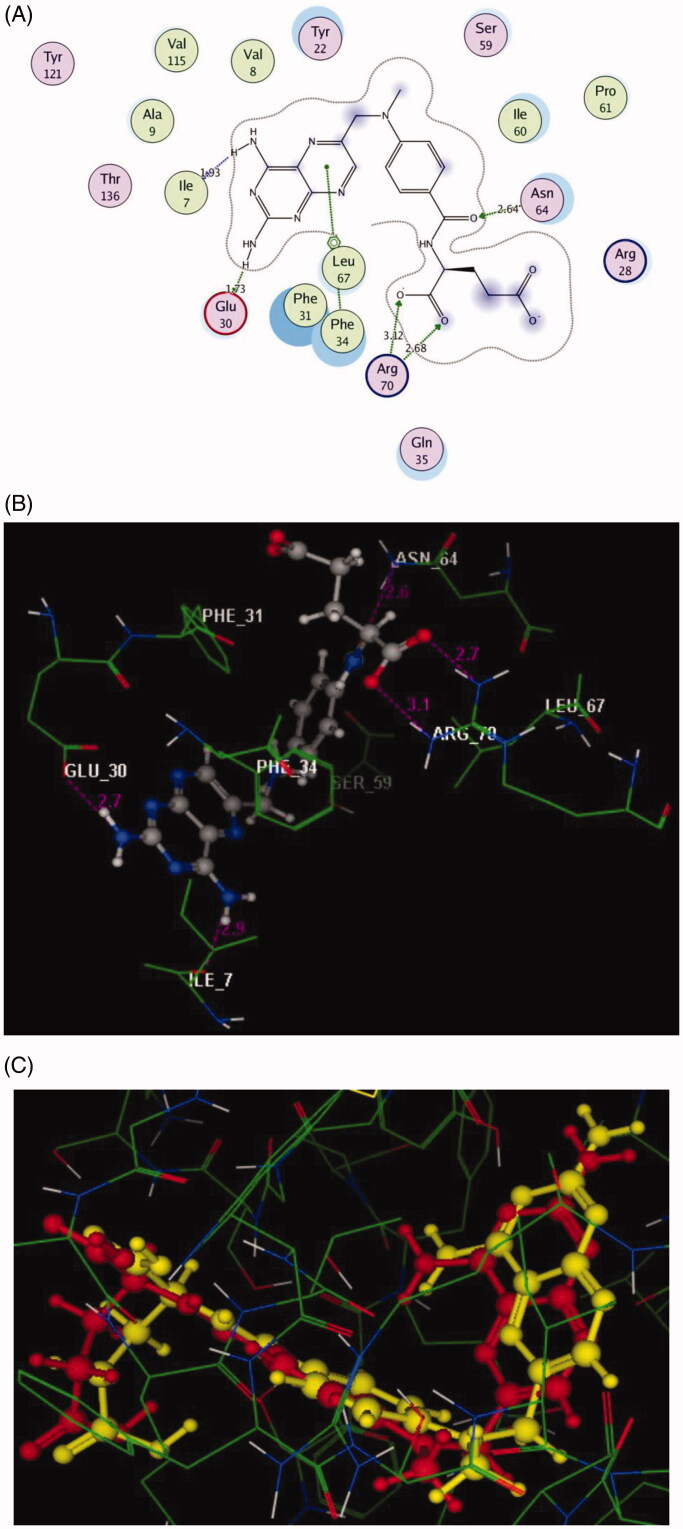
2D and 3D Views (A, B) of the original ligand, methotrexate re-docked in the active site of DHFR (PDB ID: 1DLS) using MOE software. 3D representation (C) of the superimposition of the docking pose (yellow) and the co-crystallised (red) of methotrexate with an RMSD of 0.88 Å.

In the current study, the molecular docking study was performed in order to rationalise the obtained *In vitro* enzyme assay results on dihydrofolate reductase. So, the interactions of the highly potent synthesised compounds **3a** and **6a** with the active site of DHFR were explored using MOE (Molecular Operating Environment) software 10.2008^40^^,^^41^ and through downloading of the protein data bank file (PDB ID: 1DLS)^36^ that contains the co-crystallised ligand, methotrexate. The molecular docking protocol was verified by re-docking of the original ligand in the vicinity of the active site of DHFR indicating that the docking protocol used is suitable for the intended docking study. This is shown by the score energy of −11.68 kcal/mol, the small root mean standard deviation (RMSD) between the experimental co-crystallised inhibitor pose and the docked pose of 0.88 Å and the highly noticed superimposition between them (Figure 5(c)).

The pteridine moiety of the co-crystallised ligand, methotrexate interacts with the active site of DHFR by two different interactions^42^; hydrogen bonding of the two amino groups with **Ile7** and **Glu30** (distance: 1.93 and 1.73 Å, respectively), and arene–arene interaction with **Phe34**. Moreover, the amide oxygen forms H-bond acceptor with the side chain of **Asn64** (distance: 2.64 Å), while the two oxygen of the carboxylic group shares the binding by two H-bond acceptors with **Arg70** (distance: 2.68 and 3.12 Å, respectively). This beside many hydrophobic interactions with various amino acid residues: **Ile7**, **Val8**, **Ala9**, **Tyr22**, **Arg28**, **Phe31**, **Gln35**, **Ser59**, **Ile60**, **Pro61**, **Val115**, **Tyr121**, and **Thr136**, as shown in (Figure 5).

**Figure 6.**
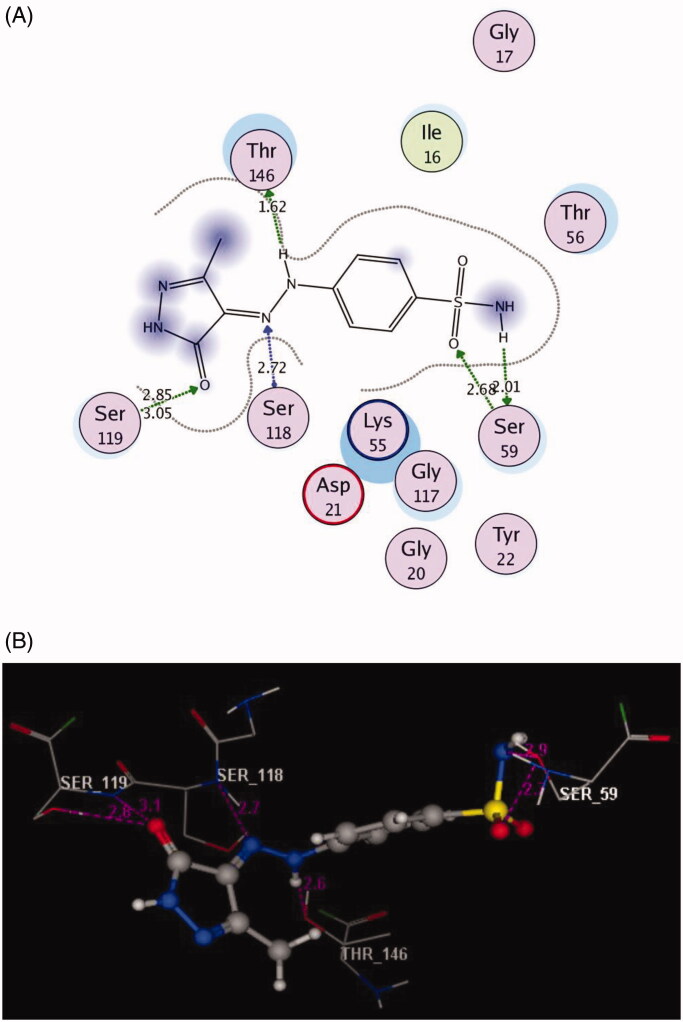
2D and 3D Views (A, B) of the compound **3a** docked in the active site of DHFR (PDB ID: 1DLS) using MOE software. Dotted lines and arrows represent hydrogen bonds.

**Figure 7.**
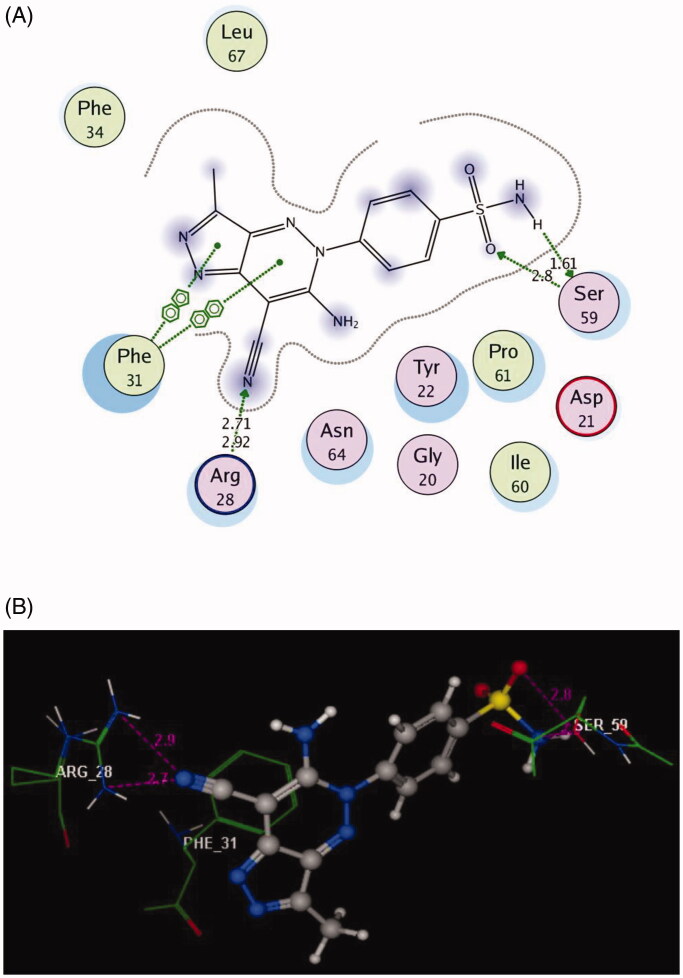
2D and 3D Views (A, B) of the compound **6a** docked in the active site of DHFR (PDB ID: 1DLS) using MOE software. Dotted lines and arrows represent hydrogen bonds.

The docked compounds **3a** and **6a** explored higher negative energy score of −12.17 and −13.48 kcal/mol indicating higher predicted binding affinity than the co-crystallised ligand. They were fit in the active site of the enzyme in a similar way via binding of the sulphonamide moiety with the side chain of **Ser59** through two hydrogen bonds (Figures 6 and 7). The two nitrogen of the hydrazinyl moiety in **3a** displayed two H-bonds, acceptor with the backbone of **Ser118** and donor with side chain of **Thr146** (distance: 2.72 and 1.62 Å, respectively). The oxygen of pyrazolone scaffold shared fixation through two H-bond acceptors with the side chain of **Ser119** (distance: 2.85 and 3.05 Å) (Figure 6).

The bulky pyrazolo[4,3-*c*]pyridazine scaffold in the compound **6a** enforced a bound conformation via two arene–arene interactions with **Phe31**. It could be assumed that chain elongation with **–NH–N=** hydrazinyl fragment between pyrazole and benzenesulfonamide in **3a**, deprived pyrazole ring from binding with the essential amino acid **Phe31**, although the insertion of **N–N** chain in cyclised ring fused to pyrazole in **6a** gave the chance to be near with **Phe31.** Furthermore, the cyano substitution on the pyazolopyridazine moiety in **6a** allowed two hydrogen bond acceptors with the side chain of **Arg28**. (distance: 2.71 and 2.92 Å, respectively) (Figure 7).

Finally, the presence of the sulphonamide substitution in the examined compounds allowed for the H-bond formation with the side chain of **Ser59** that was responsible for the observed superior inhibitory activities against DHFR. Notably, the 7-cyanopyrazolo[4,3-*c*]pyridazine substitution demonstrated excellent binding profile leading to synergistic effect.

## Conclusion

In summary, a series of pyrazole derivatives **3**, **6**, **8** and **9** bearing different heterocyclic systems was designed and synthesised. All synthesised analogues were examined for their *in vitro* antimicrobial, DHFR inhibition activity and *in silico* studies. The antimicrobial data revealed the ability of the compounds **3a** and **6a** to inhibit the growth of the screened panel of six strains with excellent MIC values in comparison with the reference drugs. In addition, the *in vitro* inhibitory activity against DHFR enzyme illustrated that compounds **3a** and **6a** were the most potent ones in comparison with methotrexate. Based on the previously obtained data from DHFR inhibition assay, the pyrazoles **3a** and **6a** containing sulphonamide substitution illustrated good fitting and favourable binding modes with DHFR enzyme in the docking study through formation of a critical hydrogen bond with the essential amino acid **Ser59**.
